# Erdheim–Chester Disease with Breast and Axillary Involvement Diagnosed by Ultrasound-Guided Biopsy: A Case Report and Literature Review

**DOI:** 10.3390/jcm15166330

**Published:** 2026-08-16

**Authors:** Juanmei Chen, Ayibota Ruxian, Danying Li, Yong Jiang, Buyun Ma

**Affiliations:** 1 Department of Ultrasound, West China Hospital, Sichuan University, Chengdu 610041, China; chenjuanmei@stu.scu.edu.cn (J.C.); ayibota_ruxian@163.com (A.R.);; 2 Department of Pathology, West China Hospital, Sichuan University, Chengdu 610041, China

**Keywords:** Erdheim–Chester disease, non-Langerhans cell histiocytosis, breast involvement, breast ultrasound, ultrasound-guided biopsy, BRAF V600E

## Abstract

**Background/Objectives**: Erdheim–Chester disease (ECD) is a rare non-Langerhans cell histiocytosis characterized by multisystem infiltration of foamy histiocytes, leading to chronic inflammation, fibrosis, and organ dysfunction. Breast involvement in ECD is extremely uncommon, and the sonographic features of ECD involving the breast remain poorly described. **Case Presentation**: We report the case of a 59-year-old woman with chronic bone pain and multisystem disease who experienced an extended diagnostic course despite undergoing renal biopsy, biopsy of a right elbow lesion, bone marrow examination, and multidisciplinary evaluation. Breast ultrasound revealed bilateral infiltrative hypoechoic lesions involving the breasts and axillae. These were classified as BI-RADS 4C and were highly suspicious for breast malignancy. Subsequently, an ultrasound-guided core needle biopsy was performed on the breast and axillary lesions. **Results**: Histopathology showed fibroadipose tissue infiltrated by numerous foamy histiocytes, scattered epithelioid cells, and occasional Touton giant cells. Immunohistochemistry showed positivity for CD68, CD163, CD4, and Cyclin D1, partial positivity for OCT2 and CD30, and negativity for S100, CD1a, Langerin, ALK, CK (Pan), and GATA3. The Ki-67 index was approximately 3%. Molecular testing detected the BRAF V600E mutation, supporting the diagnosis of ECD. A review of reported cases showed that breast involvement in ECD lacks specific ultrasound findings and may closely mimic primary breast malignancy. **Conclusions**: Breast involvement in ECD is rare and may present as bilateral infiltrative hypoechoic lesions with axillary involvement on ultrasound. In patients with chronic bone pain, symmetric osteosclerosis, or multisystem disease, ECD should be considered in the differential diagnosis. Ultrasound-detected superficial lesions may provide accessible biopsy targets, helping to establish a timely diagnosis and reduce diagnostic delay.

## 1. Introduction

Erdheim–Chester disease (ECD) is a rare non-Langerhans cell histiocytic neoplasm characterized by constitutive activation of the mitogen-activated protein kinase (MAPK) signaling pathway. Approximately 50–60% of patients harbor the BRAF V600E mutation, which is a key molecular driver of the disease [1,2]. ECD was first described by Chester in 1930 as lipoid granulomatosis and was considered clinicopathologically distinct from Hand–Schüller–Christian disease and Niemann–Pick disease [3]. In 1972, Jaffe reported a similar case and formally introduced the term Erdheim–Chester disease [4]. In 2016, ECD was included in the World Health Organization classification of hematopoietic tumors as a histiocytic neoplasm [2,5].

ECD can involve multiple organs and systems, including the skeleton, cardiovascular system, central nervous system, retroperitoneum, orbits, and skin [6]. The clinical course of ECD varies according to the extent and distribution of organ involvement, ranging from asymptomatic skeletal disease to disseminated systemic disease associated with poor prognosis [7]. Among these manifestations, symmetric osteosclerosis of the long bones accompanied by chronic bone pain is considered one of its most characteristic clinical features [8]. Breast involvement in ECD is exceptionally rare, and a 2023 review identified 16 reported patients using broad clinical and pathological inclusion criteria [9]. ECD is characterized by marked clinical heterogeneity and multisystem involvement, and diagnostic delay is common [10].

Previous reports of breast ECD have mainly focused on clinicopathology, immunophenotype, and molecular findings, including MAPK pathway activation and immune dysregulation [11,12]. Its imaging features remain poorly defined. Reported ultrasound findings vary widely, ranging from hypoechoic macrolobulated nodules and heterogeneous solid lesions with hyperechoic foci to clinically malignant masses with suspicious or indistinct margins [13,14,15,16]. These findings indicate that breast ECD lacks specific sonographic features and may mimic primary or inflammatory breast cancer. As ultrasound is central to breast lesion assessment and image-guided biopsy, better characterization of these findings is clinically important.

We present the case of a 59-year-old woman with a long history of chronic bone pain and multisystem involvement. After years of evaluation across multiple specialties, bilateral breast and axillary lesions were detected on ultrasound. Ultrasound-guided core needle biopsy provided characteristic histopathological and molecular findings, leading to the diagnosis of Erdheim–Chester disease. Furthermore, we have reviewed the available literature to summarize the sonographic features of breast involvement in ECD and to discuss the potential role of ultrasound and ultrasound-guided biopsy in supporting the diagnosis of systemic ECD.

## 2. Case Report

**Figure 1:**
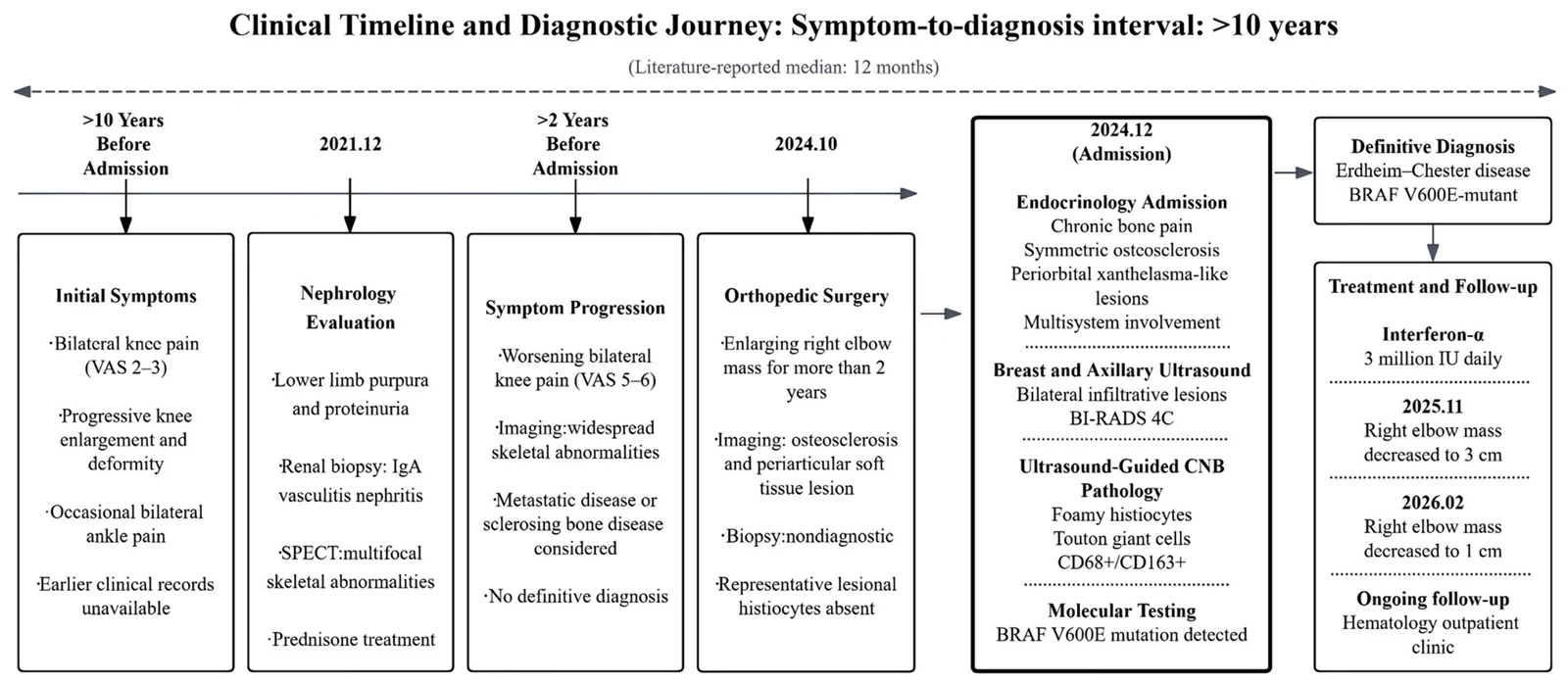
Clinical timeline from the first relevant symptom to the diagnosis and follow-up of ECD. The patient developed bilateral knee pain more than 10 years before admission in December 2024, but the exact onset date and details of early knee-related evaluation and treatment were unavailable. The symptom-to-diagnosis interval therefore exceeded 10 years, substantially longer than the literature-reported median of 12 months. CNB: core needle biopsy.

A 59-year-old woman with a history of type 2 diabetes mellitus and hypothyroidism presented to the department of endocrinology at our hospital in December 2024. She had experienced bilateral knee pain for more than 10 years and progressive multisystem symptoms. Before this visit, she had been evaluated by several departments for different organ-related conditions, but no definite diagnosis had been established. The key clinical events are summarized chronologically in Figure 1.

More than 10 years earlier, the patient developed bilateral knee pain. The pain usually occurred after prolonged standing or physical activity, with a visual analogue scale (VAS) score of 2–3. She had no pain in other joints and no fever. Over time, both knees gradually became enlarged and deformed. She also had occasional pain in both ankles. No specific diagnosis or treatment was given at that time. More than two years before the current admission, the knee pain worsened. She had difficulty standing up after squatting, and the pain was severe during squatting, with a VAS score of 5–6. There was no low back pain or radiating pain, and the symptoms improved after rest. Bone marrow aspiration from the left tibia showed active marrow proliferation, with granulocytic cells accounting for 54.3%, erythroid cells for 25%, and a mildly increased proportion of plasma cells at 7%. She was treated with vitamin D and calcium carbonate.

**Figure 2:**
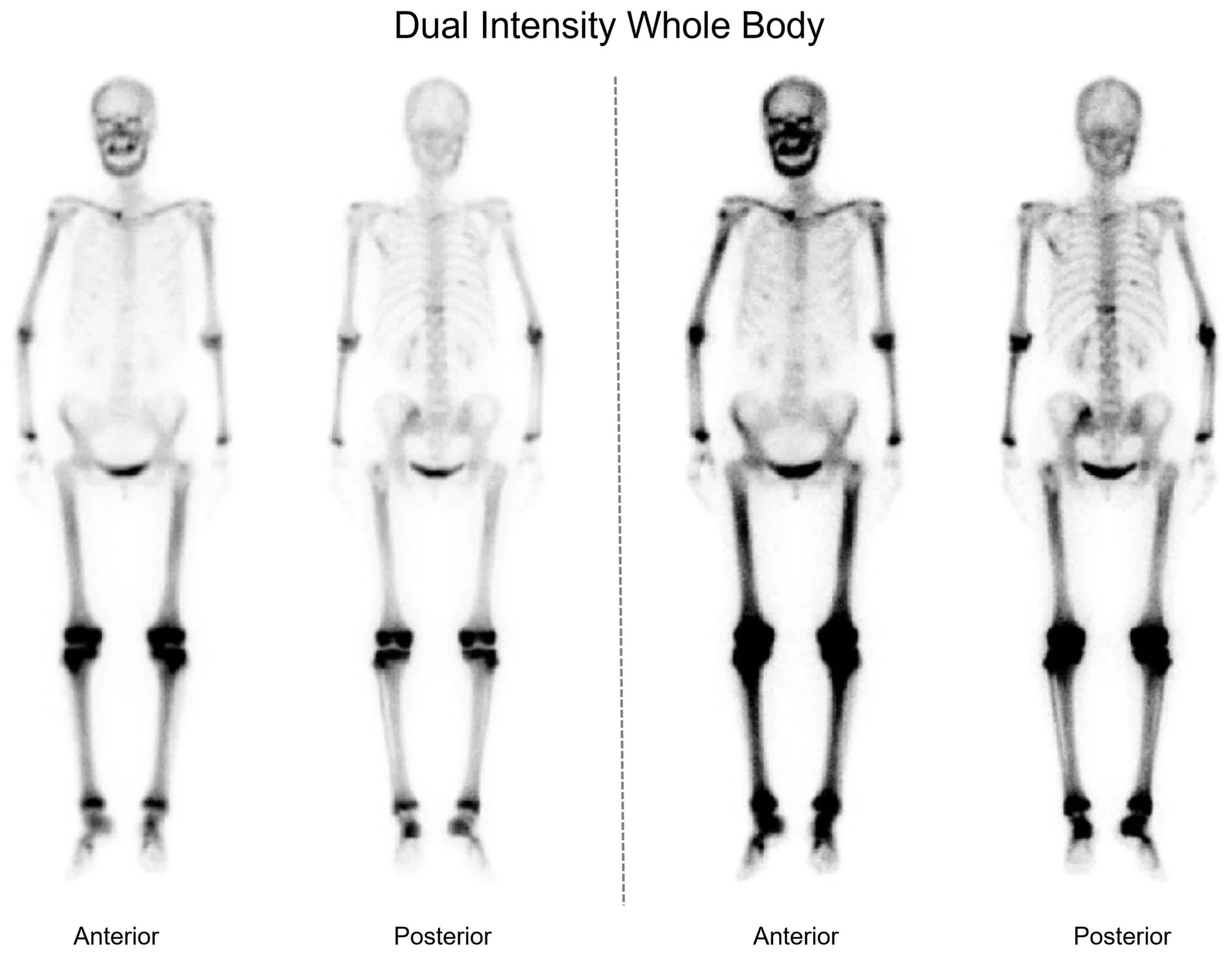
Dual-intensity whole-body 99mTc-MDP bone scintigraphy. Anterior and posterior images are presented using two display-intensity settings.Intense, bilateral, and symmetric radiotracer uptake is observed in the distal femora and proximal tibiae, with additional involvement of the distal tibiae.

In December 2021, she visited the department of nephrology because of recurrent lower-limb purpura and proteinuria. Renal biopsy confirmed IgA vasculitis nephritis. She received prednisone acetate at 30 mg once daily, which was gradually tapered and discontinued in December 2022. During the same period, chest CT at our hospital showed a mass adjacent to the descending aorta in the left lower lung region and heterogeneous increased bone density in the sternum. Subsequent whole-body SPECT bone scintigraphy showed abnormally increased uptake in the right clavicular head, left scapula, multiple right ribs, several thoracic vertebrae, and pelvis. Diffuse and symmetric increased uptake was also noted in the skull and appendicular skeleton. These findings suggested metastatic disease, metabolic bone disease, or diffuse sclerotic bone disease, but no definite diagnosis was made (Figure 2).

**Figure 3:**
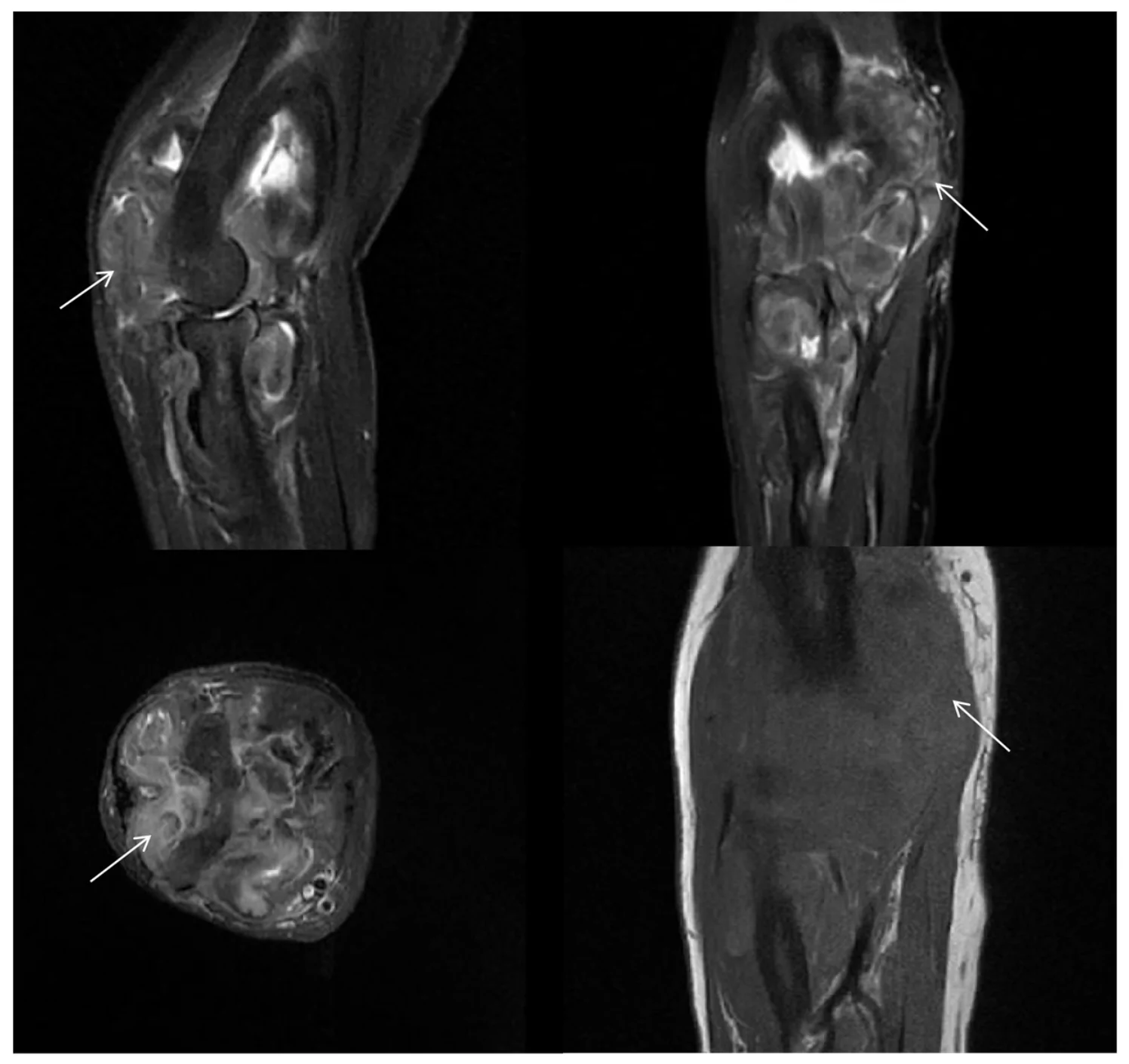
MRI findings of the right elbow lesion. Multiplanar MRI demonstrates an ill-defined infiltrative periarticular soft-tissue lesion around the right elbow, with heterogeneous signal intensity and adjacent osseous erosion. The arrows indicate the location of the lesion.

In October 2024, the patient was admitted to the orthopedic ward for evaluation of a progressively enlarging mass around the right elbow, which had been present for more than 2 years. Radiography showed focal osteosclerosis. MRI suggested an infiltrative periarticular soft tissue lesion with adjacent cortical bone erosion (Figure 3). The imaging differential diagnosis included pigmented villonodular synovitis and synovial tumor. Open biopsy of the right elbow lesion was then performed. Pathological examination showed predominantly skeletal muscle and fibroadipose tissue with a few thick-walled vessels, but no convincing histiocytic infiltrate. Because representative lesional histiocytes were absent, no informative ECD-specific immunohistochemical results could be obtained from this specimen, and the biopsy remained nondiagnostic.

At the endocrinology visit in December 2024, physical examination revealed bilateral periorbital xanthelasma-like lesions, a palpable mass around the right elbow, bilateral axillary lymphadenopathy, and mild swelling of both knees. Given the combination of chronic bone pain, multifocal osteosclerosis, and multisystem involvement, a systemic histiocytic disorder was suspected. Breast and axillary ultrasound was performed to evaluate the enlarged axillary lymph nodes.

**Figure 4:**
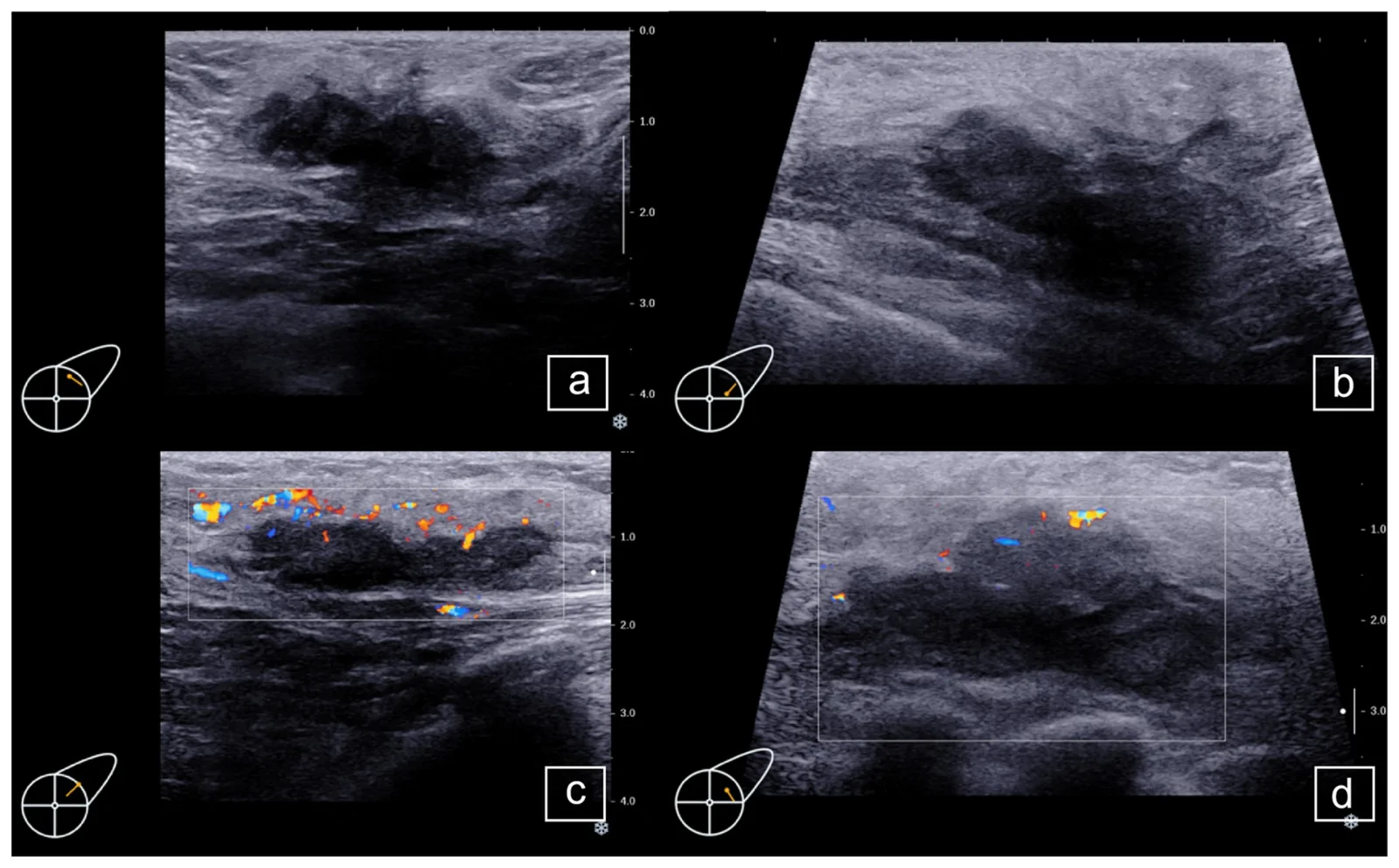
Ultrasound images of left breast masses. (**a**,**c**) Grayscale and color Doppler ultrasound of the irregular hypoechoic breast mass at the margin of the glandular layer in the 1 o’clock position of the left breast, with internal and peripheral vascularity. (**b**,**d**) Grayscale and color Doppler ultrasound of the infiltrative hypoechoic lesion with indistinct margins at the margin of the glandular layer in the 2 o’clock position of the left breast, with internal and peripheral vascularity. The white rectangular boxes in panels (**c**,**d**) indicate the color Doppler regions of interest used to display blood-flow signals.

Ultrasound showed heterogeneous infiltrative hypoechoic lesions in both breasts and axillary regions. In the left breast, irregular hypoechoic masses measuring approximately 34 × 14 × 29 mm and 48 × 18 × 34 mm were detected at the 1 o’clock (Figure 4a,c) and 2 o’clock (Figure 4b,d) positions, respectively. Both lesions had indistinct margins and irregular shapes. They extended anteriorly into the subcutaneous layer, and the posterior border with the retromammary space was unclear. Patchy hyperechoic areas were present within parts of the lesions. Punctate and linear blood flow signals were seen within and around the masses, with arterial waveforms detected on Doppler imaging.

**Figure 5:**
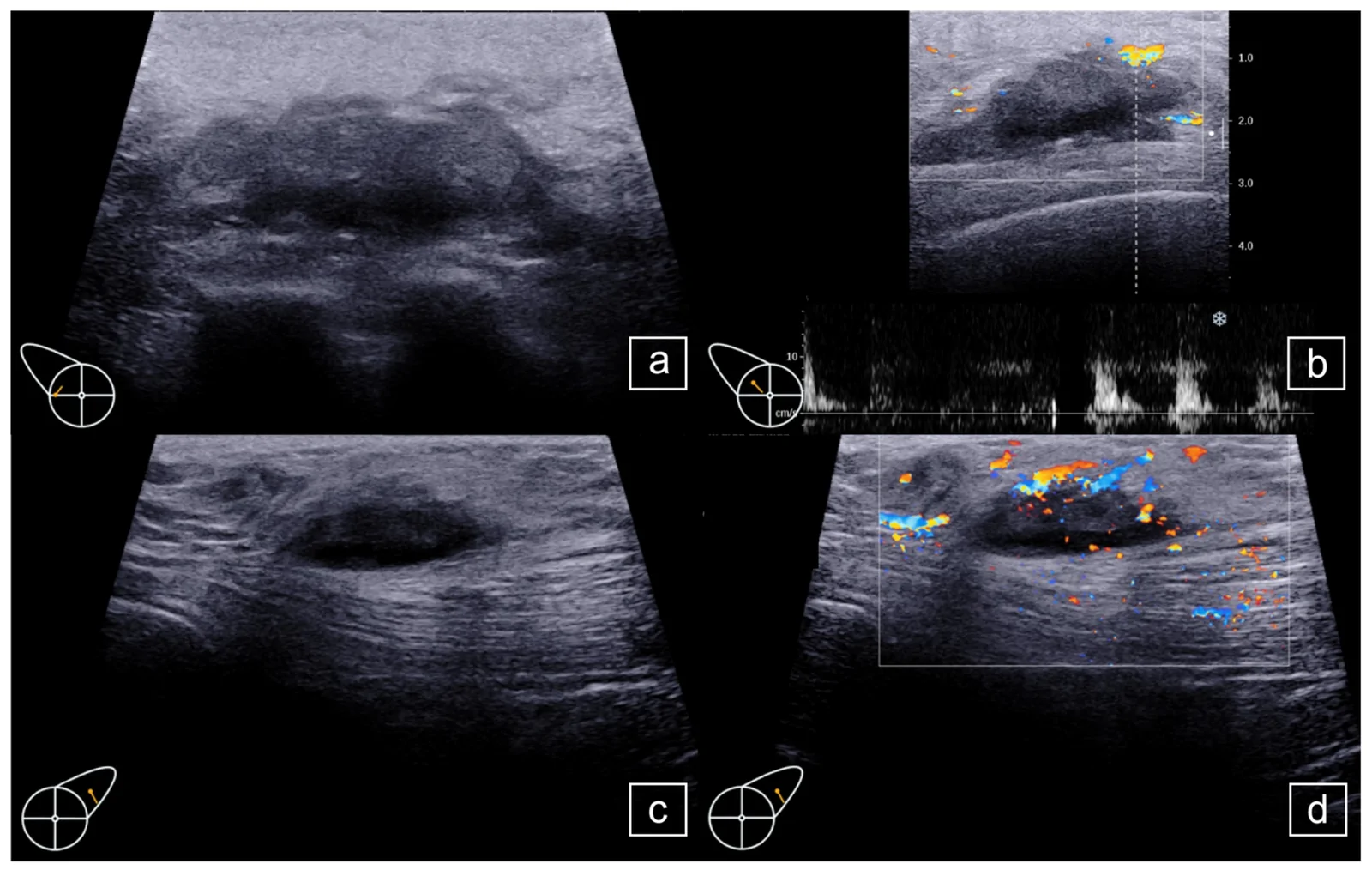
Ultrasound findings of right breast and bilateral axillary involvement. (**a**,**b**) Grayscale and color Doppler ultrasound of the lateral right breast extending to the axilla showing contiguous infiltrative hypoechoic masses with indistinct margins, irregular morphology, patchy internal hyperechoic areas, and internal and peripheral vascularity. (**c**,**d**) Grayscale and color Doppler ultrasound of the left axilla showing an irregular hypoechoic mass with indistinct margins and internal and peripheral vascularity. The white rectangular boxes in panels (**b**,**d**) indicate the color Doppler regions of interest, while the dashed line in panel (**b**) represents the Doppler sampling line used to obtain the spectral waveform.

In the lateral right breast, several contiguous hypoechoic masses in the subcutaneous and glandular layers extended superolaterally into the right axillary region, with a maximum thickness of approximately 60 mm. These lesions had indistinct margins, irregular shapes, patchy internal hyperechoic areas, and internal and peripheral Doppler flow signals (Figure 5a,b). A separate hypoechoic soft-tissue mass measuring approximately 27 × 9 × 20 mm was identified in the left axilla (Figure 5c,d). Several lymph nodes were also identified in both axillae. The largest measured approximately 18 × 5 mm on the left and 12 × 8 mm on the right. Some nodes showed an indistinct corticomedullary junction and punctate or linear internal blood flow. Based on their irregular morphology, infiltrative appearance, and vascular features, the bilateral breast lesions with right breast-to-axillary extension and the separate left axillary mass were classified as BI-RADS category 4C and were considered suspicious for malignancy.

**Figure 6:**
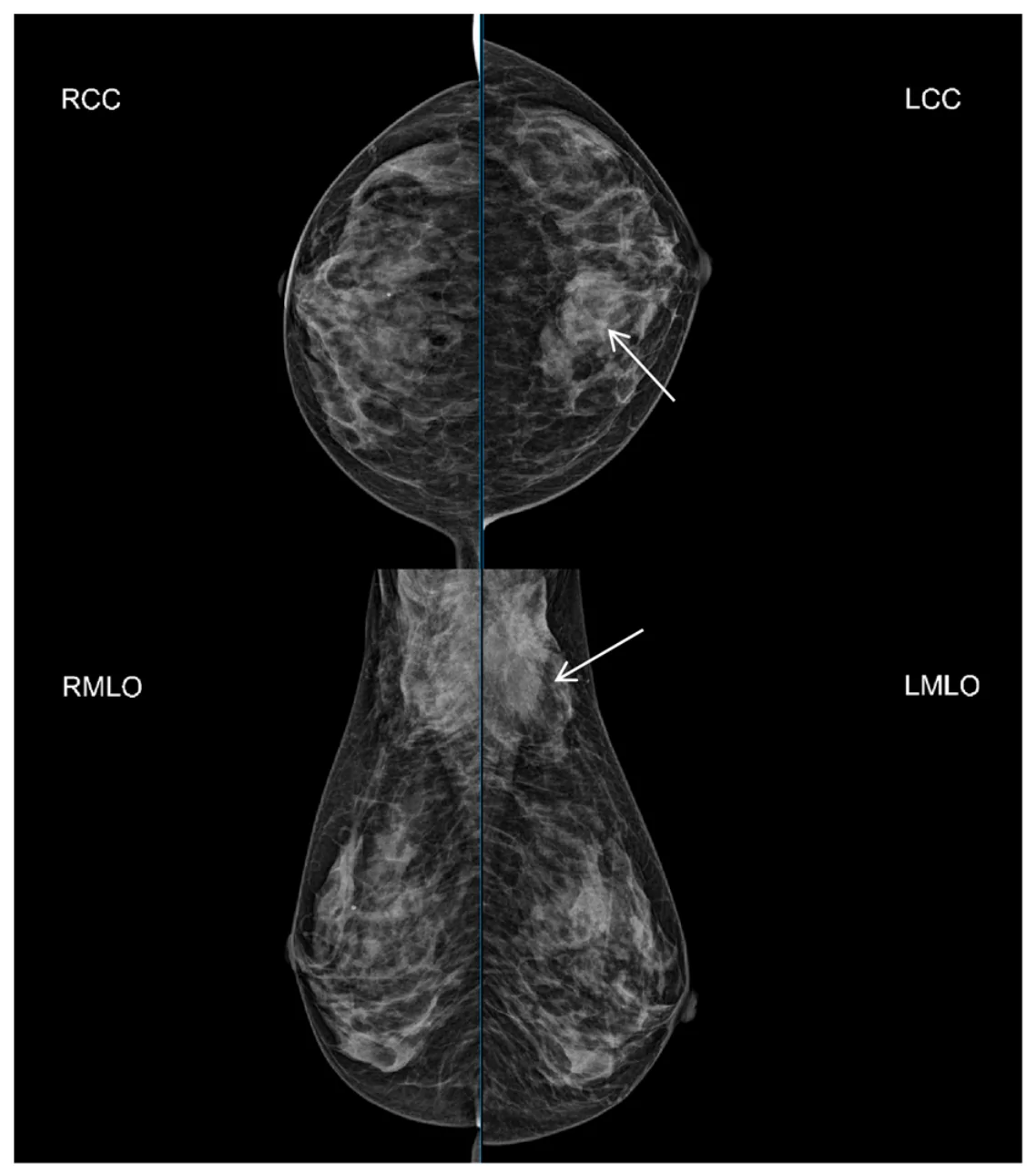
Mammographic findings of breast and axillary involvement. Bilateral digital mammography in craniocaudal and mediolateral oblique views showed diffuse increased breast density with heterogeneous patchy opacities and blurred margins. The arrows indicate the lesion sites in the left breast and left axillary region, where locally increased density and irregular soft tissue opacity were observed. RCC, right craniocaudal; LCC, left craniocaudal; RMLO, right mediolateral oblique; and LMLO, left mediolateral oblique.

Digital mammography of both breasts showed diffuse increased breast density with heterogeneous patchy opacities and blurred margins. The upper quadrant of the left breast showed locally higher density than the right side and was assessed as BI-RADS category 3. Irregular soft tissue density was also seen in both axillae. On the left side, adjacent skin thickening and retraction were noted. Bilateral accessory breast tissue was considered, and a coexisting tumor on the left side could not be excluded (Figure 6).

**Figure 7:**
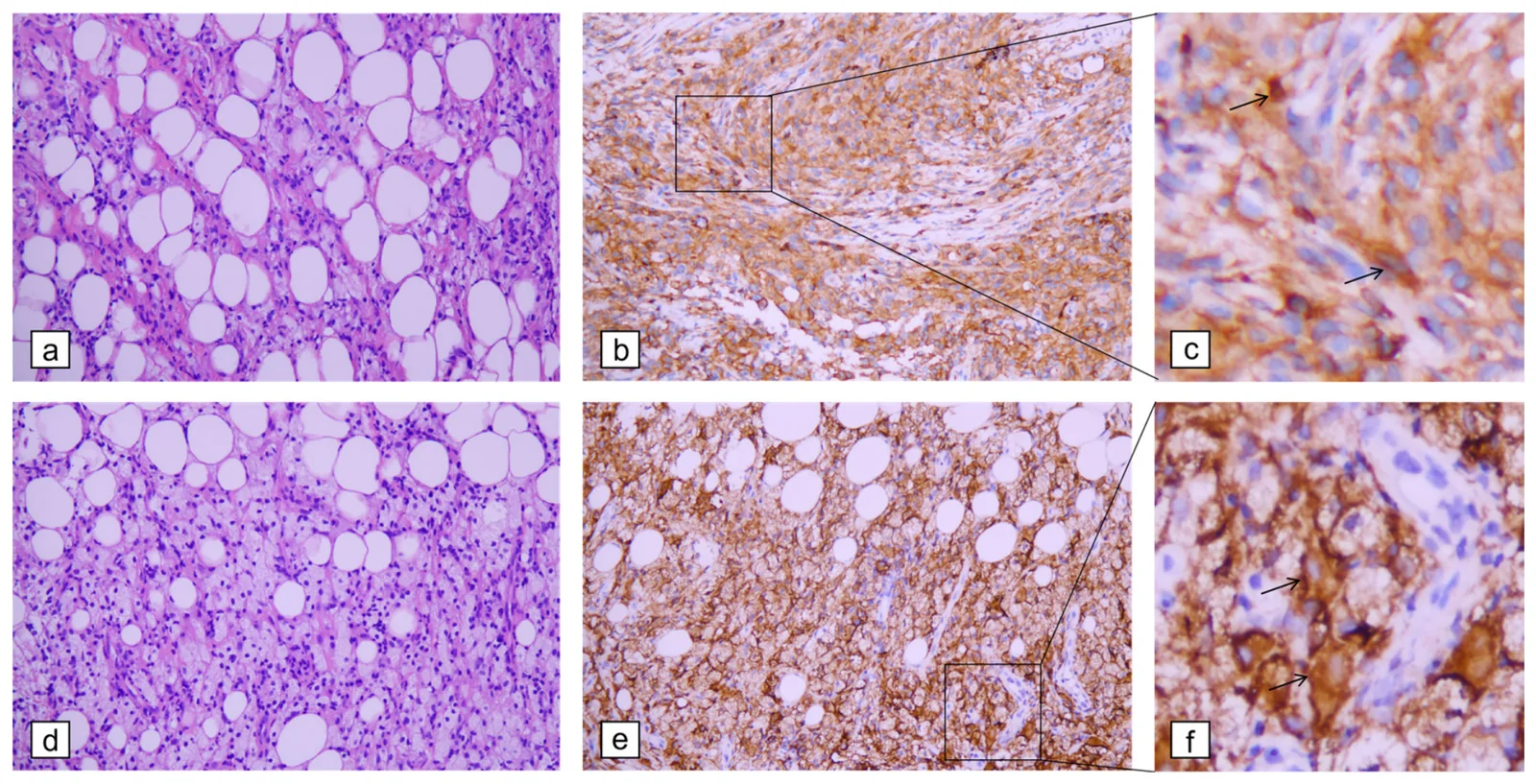
Histopathological findings of the left breast and left axillary lesions. (**a**) The left breast lesion shows infiltration of fibroadipose tissue by foamy histiocytes (H&E staining, ×200). (**b**,**c**) CD4-positive cell infiltration in the left breast lesion (CD4 immunohistochemical staining, ×200). (**d**) The left axillary lesion shows infiltration of fibroadipose tissue by foamy histiocytes (H&E staining, ×200). (**e**,**f**) CD163-positive cell infiltration in the left axillary lesion (CD163 immunohistochemical staining, ×200). The black box indicates the region of interest, which is shown at higher magnification in the right panel; the black arrows indicate representative histiocytes with distinct cytoplasmic staining.

Ultrasound-guided core needle biopsy was subsequently performed for the bilateral breast and axillary lesions. Histopathological examination showed fibroadipose tissue infiltrated by numerous foamy histiocytes and scattered epithelioid cells, with occasional Touton giant cells. Small lymphocytes and plasma cells were present in the background, either focally or in a scattered distribution. Immunohistochemistry showed that the lesional cells were positive for CD68 (PG-M1), CD163, CD4, and Cyclin D1; partially positive for OCT2 and CD30; and negative for S100, CD1a, Langerin, ALK (clone OTI1H7), CK (Pan), and GATA3 (Figure 7). The Ki-67 proliferation index was low, at approximately 3%. Further mutation testing using the ADx-ARMS assay detected the BRAF V600E mutation. KRAS, NRAS, and PIK3CA mutations were not detected. The targeted assay did not include MAP2K1 (MEK1) and its status was not assessed. Based on the morphological, immunohistochemical, and molecular findings, the diagnosis was a histiocytic neoplasm with BRAF V600E mutation, consistent with Erdheim–Chester disease.

IgG4-related disease was considered in the differential diagnosis because of the multisystem involvement and periaortic soft tissue infiltration. However, it was considered less likely because the serum IgG4 level was not elevated and characteristic histopathological features of IgG4-related disease were absent. Instead, the symmetric long-bone involvement, histiocytic immunophenotype, and BRAF V600E mutation strongly supported ECD. Tissue IgG4 immunostaining was not performed, which represents a limitation of this case.

After years of evaluations across multiple specialties, a definitive diagnosis was finally established. The patient was referred to the Department of Hematology for systemic treatment and long-term follow-up. Interferon-α therapy was started at a dose of 3 million international units daily. Supportive treatment was also provided for hypothyroidism, metabolic abnormalities, and chronic pain. Serial follow-up showed a favorable clinical response. In November 2025, the maximum diameter of the right elbow mass decreased to approximately 3 cm. By February 2026, it had further decreased to approximately 1 cm. No clinically apparent new organ involvement was documented during the available follow-up, and the patient remained clinically stable under multidisciplinary care.

## 3. Discussion

Erdheim–Chester disease (ECD) is a rare non-Langerhans cell histiocytosis with frequent multisystem involvement and highly heterogeneous clinical manifestations [17]. ECD lesions accumulate in the affected organs and tissues, and spontaneous degeneration is relatively rare [18]. The present case illustrates several diagnostic challenges that are typical of ECD. The patient had chronic bone pain, multifocal osteosclerosis, periorbital xanthelasma-like lesions, periarticular soft-tissue disease, and breast and axillary lesions. These findings were assessed separately by different specialties over several years. The diagnosis was established only after ultrasound-guided biopsy of superficial breast and axillary lesions provided adequate tissue for histopathological, immunohistochemical, and molecular evaluation. This diagnostic course highlights the importance of integrating apparently unrelated clinical and imaging findings into a single systemic disease framework when ECD is suspected.

The extended diagnostic course in this patient reflects the rarity and variable organ involvement of ECD. Previous studies reported a median interval of 12 months from symptom onset to diagnosis and a median of two biopsies before a definitive diagnosis [10]; in the present case, the interval from initial bilateral knee pain to diagnosis exceeded 10 years. Dense fibrosis in long-standing lesions, limited biopsy sampling, and prior corticosteroid or immunosuppressive therapy may contribute to the paucity of typical lesional histiocytes, thereby complicating pathological diagnosis [19]. Compared with the later breast and axillary specimens, which contained numerous foamy histiocytes, scattered epithelioid cells, and occasional Touton giant cells, the elbow specimen was markedly less informative and consisted predominantly of skeletal muscle and fibroadipose tissue without a diagnostic histiocytic population. The nondiagnostic result may therefore reflect sampling of a fibrotic, low-cellularity, or peripheral region; the patient’s prednisone exposure from 2021 to 2022 may also have contributed, although this cannot be established retrospectively. Consensus recommendations favor biopsy of metabolically active and readily accessible lesions whenever feasible [1,2]. In this patient, the superficial breast and axillary lesions were clearly visualized on ultrasound and yielded cellular, representative tissue for histopathological, immunohistochemical, and molecular evaluation, underscoring the importance of biopsy-site selection.

Breast involvement in ECD is rare. As ultrasonography remains the primary imaging modality for breast disease evaluation, characterization of the sonographic features of breast ECD is of considerable clinical importance. We performed a targeted review of PubMed and Web of Science up to 17 May 2026, using the terms “Erdheim-Chester” and “breast” or “breasts”, supplemented by manual screening of reference lists. Only English-language reports with histopathologically confirmed ECD involving the breast and available breast ultrasonographic descriptions were included. Seven published cases were identified and are summarized together with the present case in Table 1. In contrast, Giardino et al. applied broader inclusion criteria and summarized 16 patients with breast involvement by ECD. Their review did not require every patient to have available breast ultrasonographic findings or histopathological confirmation based on breast tissue specimens [9].

The available literature indicates that breast involvement in ECD most commonly appears as suspicious malignant-appearing solid lesions on ultrasonography, although the sonographic features are variable and lack specificity. Reported findings include unilateral or bilateral breast masses, multifocal lesions, hypoechoic or heterogeneous echogenicity, irregular or lobulated morphology, indistinct margins, posterior acoustic attenuation or enhancement, and variable vascularity [13,14,15,16,20,21,22]. These imaging features often overlap with those of primary breast carcinoma, inflammatory breast carcinoma, metastatic disease, lymphoma, or granulomatous inflammatory lesions.

Compared with previously reported cases, the present case demonstrated a more extensive infiltrative pattern involving both breasts and axillary regions. The lesions were bilateral, multifocal, irregular, and hypoechoic, with indistinct margins, patchy internal hyperechoic foci, and detectable internal or peripheral vascularity. Multiple confluent hypoechoic lesions in both breasts formed large infiltrative masses, with arterial flow spectra detected within and around the lesions. These findings resulted in a BI-RADS 4C assessment and closely mimicked breast malignancy. Histopathologically, the diffuse infiltration of fibroadipose tissue by numerous foamy histiocytes and scattered epithelioid cells provided an explanation for the infiltrative hypoechoic appearance and poorly defined margins on ultrasound.

This case expands the known ultrasonographic spectrum of breast involvement in ECD. It suggests that breast ECD may present not only as relatively circumscribed nodular lesions, but also as aggressive malignant-appearing infiltrative lesions with axillary involvement. Therefore, bilateral or multifocal distribution should not lead to a downgrade in suspicion. When breast or axillary lesions show suspicious features—including irregular morphology, indistinct margins, infiltrative growth, vascularity, skin or subcutaneous extension, or axillary involvement—tissue biopsy should be pursued according to BI-RADS assessment. For patients with chronic bone pain, symmetric osteosclerosis, xanthelasma-like lesions, or other unexplained multisystem manifestations, ECD should be considered as part of the differential diagnosis.

Skeletal involvement is one of the most common clinical manifestations of ECD. It usually presents as bone pain, especially around the knees and ankles. The characteristic imaging finding is bilateral, symmetric osteosclerosis of the metadiaphyseal regions of the long bones, predominantly around the knees [23]. This is a classic diagnostic feature of ECD and can be detected by radiography, CT, bone scintigraphy, or PET/CT [24]. Retroperitoneal and urinary involvement in ECD may include perirenal infiltration, the hairy kidney sign, renal sinus or ureteral involvement, and hydronephrosis [25]. In this patient, urinary ultrasonography in December 2021 showed bilateral hydronephrosis, but the history of proteinuria and renal biopsy findings complicated the diagnosis and may have masked an underlying systemic histiocytic disorder. Cardiovascular involvement may include periaortic encasement, coronary or renal artery involvement, right atrial pseudotumor, and pericardial disease [26]. In this case, the mass adjacent to the descending aorta was considered compatible with periaortic or paravertebral soft-tissue involvement. Ocular and orbital manifestations are also important diagnostic clues. Common findings include proptosis, orbital involvement, eyelid involvement, and periorbital xanthelasma-like lesions [27,28]. In this patient, the bilateral periorbital xanthelasma-like lesions remained clinically meaningful, even though no definite retro-orbital mass was identified.

In retrospect, the patient’s longstanding bone pain, abnormal bone scintigraphy findings, and periorbital xanthelasma-like lesions were all compatible with systemic ECD. So too were the soft-tissue masses and breast and axillary abnormalities. Current consensus recommendations advise comprehensive baseline evaluation in suspected ECD, including whole-body FDG-PET/CT, brain MRI, cardiac MRI, contrast-enhanced chest, abdominal, and pelvic CT; laboratory and endocrine assessment should also be performed to define the full extent of disease involvement [2]. In this patient, whole-body FDG-PET/CT was not performed, and the lack of an integrated systemic assessment may have contributed to the extended diagnostic course. Moreover, the actual extent and distribution of multisystem involvement, along with baseline staging, prognostic assessment, and the metabolic activity of potential biopsy sites, remained incompletely characterized. This also precluded comparison of treatment responses across different organs.

Genotype may contribute to organ tropism in ECD. Reported genotype–phenotype associations link BRAF mutations with neurological, pituitary, and cardiovascular involvement; KRAS and NRAS mutations predominantly with cutaneous and pleural involvement, respectively, and MAP2K1 mutations with peritoneal and retroperitoneal disease [29]. Among the eight breast ECD cases summarized in Table 1, five were reported as BRAF-positive (including four with explicitly BRAF V600E and one with the specific variant not reported), one was BRAF V600E-negative, and two did not report BRAF testing. This distribution raises the hypothesis that breast or axillary involvement may be enriched in BRAF-altered ECD; however, the very small number of selectively reported cases precludes concluding that breast involvement is a genotype-dependent phenotype.

ECD is now regarded as a clonal inflammatory myeloid neoplasm driven mainly by activation of the MAPK/ERK signaling pathway [18]. BRAF V600E is the most frequent and clinically important driver mutation [12]. MAP2K1 is another recurrent MAPK pathway alteration, particularly in BRAF wild-type ECD, and should be included in broader molecular profiling when feasible [2,18]. In this patient, MAP2K1 testing was not performed because the available targeted assay did not include this gene. This limits complete molecular characterization. BRAF V600E was identified as a clear driver and detection of BRAF V600E has direct therapeutic implications. The 2020 consensus recommendations emphasized molecular testing in ECD and noted that BRAF inhibitors may be considered for patients with BRAF V600-mutant disease [2]. Targeted therapy for ECD has recently emerged as an important therapeutic strategy. For patients with BRAF V600-mutant ECD, BRAF inhibitors—with or without MEK inhibitors—have become important mutation-directed treatment options. Treatment decisions should be individualized according to disease severity, organ involvement, drug availability, toxicity profile, and patient preference [30].

However, conventional therapy may still be considered in selected patients. The 2020 consensus recommendations allow either BRAF inhibition or conventional therapy for BRAF V600E-mutant ECD without end-organ dysfunction [2]. In the available assessments for this patient, no critical central nervous system or cardiac involvement or other immediately life-threatening end-organ dysfunction was documented. After multidisciplinary evaluation and shared decision-making, interferon α (IFN-α) was selected because of the apparent organ-risk profile, drug availability, financial considerations, and patient preference. IFN-α and pegylated interferon α (PEG-IFN-α) are established conventional therapies for ECD, with reported efficacy rates ranging from 50% to 80% [2], and PEG-IFN-α has shown favorable disease control and manageable toxicity [31]. IFN-α was initiated at 3 million IU daily, followed by a marked decrease in the right elbow mass from approximately 3 cm in November 2025 to 1 cm in February 2026. This response was documented only at the elbow. Because post-treatment ultrasonography of the breast and axillary lesions was not performed, the response of those lesions is unknown, and the present case cannot establish the general applicability of ultrasound for treatment monitoring. Continued systemic and site-specific follow-up remains necessary, particularly given the incomplete baseline FDG-PET/CT staging.

This case highlights that superficial breast and axillary lesions may serve not only as important sites of disease involvement but also as readily accessible targets for diagnostic tissue sampling in systemic ECD. Ultrasound plays a pivotal role in identifying and guiding biopsy of these lesions, enabling acquisition of representative tissue for histopathological and molecular analyses. In patients with unexplained multisystem disease, ultrasound evaluation and ultrasound-guided biopsy may facilitate earlier diagnosis, reduce diagnostic delay, and help integrate seemingly disparate clinical findings into a unifying diagnosis.

## 4. Conclusions

Breast involvement is a rare manifestation of Erdheim–Chester disease (ECD). On ultrasonography, it may present as bilateral, multifocal, infiltrative hypoechoic lesions with indistinct margins, irregular morphology, and associated axillary involvement, closely mimicking breast malignancy and posing a significant diagnostic challenge. ECD should therefore be considered in the differential diagnosis of patients with chronic bone pain, symmetric osteosclerosis, periorbital xanthelasma-like lesions, or other manifestations of multisystem involvement.

## Figures and Tables

**Table 1:** Summary of sonographic and pathological characteristics of breast involvement in Erdheim–Chester disease (ECD) reported in the literature.

Author/Year	Patient	Site of Lesions	Size	Morphology	Margin	Echogenicity	Posterior Feature	Blood Flow	BI-RADS Category	Impression	Key Pathology	BRAF Status
Provenzano et al. 2010 [16]	A 78-year-old female	Bilateral breast masses	Left 56 mm Right 53 mm	Irregular infiltrative	Ill-defined	Heterogeneous hypoechoic	Posterior attenuation	NR	NR	Bilateral locally advanced breast cancer	Diffuse foamy histiocytic infiltrate, scattered Touton giant cells, CD68(+), CD1a(−), S100(−)	NR
Basara et al. 2015 [13]	A 62-year-old female	Multiple, bilateral breast + axillary masses	NR	Irregular macrolobulated	NR	Hypoechoic	NR	NR	BI-RADS 4 lesions	Suspicious for malignant	Foamy histiocytes, CD68(+), S100(−)	NR
Guo et al., 2015 [20]	A 61-year-old female	Single right breast mass	3 × 2 cm	Unencapsulated	NR	Hypoechoic	NR	NR	NR	Clinically malignant tumor	Foamy histiocytes, Touton giant cells, fibrosis, CD68(+), CD1a(−), S100(−)	BRAF V600E(+)
Binyousef et al. 2017 [15]	A 52-year-old female	Multiple (bilateral breast + bilateral axillary masses)	Large	Irregular Lobulated infiltrative	NR	Hypoechoic	Posterior attenuation	Increased vascularity	BI-RADS 4	indeterminate	Xanthomatous histiocytes, CD68(+), CD1a(−), S100(−)	BRAF V600E(+)
Sprenger et al., 2024 [14]	A 79-year-old female	Multiple, bilateral breast masses	NR	Oval Lobulated horizontalized coalescing	Blurred	Heterogeneous with internal hyperechoic foci	Posterior attenuation	NR	NR	NR	Infiltrating foamy histiocytes, CD68(+), CD1a(+)	BRAF V600E(−)
Jia et al., 2024 [21]	A 40-year-old female	Single right breast	NR	NR	Well-defined	Hypoechoic area	NR	NR	NR	NR	Histiocytes, giant cells; CD68(+), CD1a(−), S100(−)	BRAF V600E(+)
Oska & Parikh, 2025 [22]	A 45-year-old female	Multiple, bilateral breast masses	NR	Irregular spiculated	fingerlike projections	Hypoechoic	NR	With associated vascularity	High suspicion for malignancy	Mimicked inflammatory breast carcinoma	Diffuse lipidized histiocytosis, Touton giant cells, CD68(+), CD1a(−)	BRAF(+)
The present case	A 59-year-old female	Multiple (bilateral breast + bilateral axillary masses)	Left breast 48 × 18 × 34 mm Right breast thickness 60 mm	Irregular Infiltrative confluent	Ill-defined	Hypoechoic with patchy hyperechoic foci	NR	Internal and peripheral linear blood flow, arterial spectrum	BI-RADS 4C	Bilateral breast and axillary solid masses, suspicious for carcinoma	Foamy histiocytes, epithelioid cells, CD68(+), CD1a(−), S100(−)	BRAF V600E(+)

Abbreviations: NR, not reported. Only English-language cases with histopathologically confirmed ECD involving the breast and available breast ultrasonographic descriptions were summarized.

## Data Availability

The original contributions presented in this study are included in the article. Further inquiries can be directed to the corresponding authors.

## Abbreviations

The following abbreviations are used in this manuscript:

ECD	Erdheim–Chester disease
BI-RADS	Breast Imaging Reporting and Data System
CNB	Core needle biopsy
H&E	Hematoxylin and eosin
IU	International unit
